# Endothelial Activation and Microcirculatory Disorders in Sepsis

**DOI:** 10.3389/fmed.2022.907992

**Published:** 2022-06-03

**Authors:** Lisa Raia, Lara Zafrani

**Affiliations:** ^1^Medical Intensive Care Unit, Hôpital Saint-Louis, Assistance Publique des Hôpitaux de Paris, Paris, France; ^2^INSERM UMR 976, University of Paris Cité, Paris, France

**Keywords:** endothelial dysfunction, sepsis, microcirculation, organ failure, peripheral perfusion, endothelium

## Abstract

The vascular endothelium is crucial for the maintenance of vascular homeostasis. Moreover, in sepsis, endothelial cells can acquire new properties and actively participate in the host's response. If endothelial activation is mostly necessary and efficient in eliminating a pathogen, an exaggerated and maladaptive reaction leads to severe microcirculatory damage. The microcirculatory disorders in sepsis are well known to be associated with poor outcome. Better recognition of microcirculatory alteration is therefore essential to identify patients with the worse outcomes and to guide therapeutic interventions. In this review, we will discuss the main features of endothelial activation and dysfunction in sepsis, its assessment at the bedside, and the main advances in microcirculatory resuscitation.

## Introduction

Sepsis is a life-threatening condition defined by multi-organ dysfunction secondary to a dysregulated host response to infection (1). Incidence of sepsis represents one of the leading causes of hospitalization in the Intensive Care Unit (ICU) and mortality remains high despite several improvements in early resuscitation (2).

The vascular endothelium consists of a single cell layer at the interface of the circulating blood and vessel wall. Composed of about 10^13^ cells representing 1.5 kg, the endothelium maintains microvascular homeostasis, regulating vascular tone, primary hemostasis, and cellular traffic. By its privileged localization, the vascular endothelium plays a crucial role in the response to infection. However, the exaggerated host response can cause structural and functional endothelial damage. Thus, most endothelial functions are disrupted in sepsis, leading to microthrombi, tissue edema, interstitial leakage, and dysregulated vascular tone.

Experimental and clinical studies have evidenced microcirculatory abnormalities in sepsis, which are strongly associated with organ dysfunction and mortality (3). The correction of systemic hemodynamic variables fails to restore microcirculatory perfusion and suggests a need for new axes and new therapeutics.

In this review we will discuss the key role of the endothelium in the microvascular response to sepsis.

## Vascular Endothelium

### Structure

The vascular endothelium, comprising a cell monolayer, covers the interior surface of blood vessels all along the vasculature. About 10^1^3 endothelial cells (ECs), covering 1000 m^2^, provide a direct interface between the circulating blood cells and the vessel wall. ECs share common properties but are also heterogeneous according to specific organs and vascular beds, both concerning their structure and their function (4). This also implies distinct responses to pathological conditions with various structural and functional modifications.

At their surface, ECs are covered by a multicomponent layer, the glycocalyx, consisting of proteoglycans, glycoprotein, and glycosaminoglycans (5).The glycocalyx constitutes a first-line barrier which provides the regulation of cellular and molecular traffic. In addition, because of its negative electrical charge, the glycocalyx acts as an anticoagulant layer. The glycocalyx participates in vascular homeostasis as a vascular barrier, powerful antioxidant, and transducer of shear stress to the endothelium (6).

Regarding its weight and its numerous functions, the endothelium should be considered as a fully-fledged organ.

### Resting Endothelium

The integrity of ECs is a chief regulator of vascular homeostasis. The vascular endothelium has a fundamental role in several physiologic processes such as vasomotor tone regulation, primary hemostasis, osmotic balance, and vascular barrier function. ECs also perform important immunologic functions. By sensing pathogen components present in blood, ECs can initiate the immune response. ECs are also conditional antigen-presenting cells (APCs) and, in some specific situations, allow the initiation of adaptive immunity (7). In normal conditions, ECs do not interact with circulation leukocytes, mainly because of the glycocalyx barrier and internalized membrane adhesion molecules.

Primary hemostasis is the process in which platelets adhere, activate, and aggregate to restore vascular integrity after an aggression. The endothelium synthesizes and expresses key hemostasis regulating factors, such as the von Willebrand factor (vWF) and tissue factor (TF). When the endothelium is damaged, the vWF is exposed to the circulation and can initiate platelet recruitment to the lesion. Then, binding to glycoproteins GPIa, GPVI, GPIb-IX-V, platelets activate and can enhance recruitment and activation of circulating platelets in order to form a clot.

At rest, the endothelium has anticoagulant and profibrinolytic properties. While the endothelium is one of the main producers of TF, it also negatively regulates the TF pathway, producing the TF pathway inhibitor (TFPI). TFPI limits thrombin generation, binding to activated factor X and inhibiting aFVII complex. In addition, ECs are responsible for thrombomodulin (TM) production and release. TM, combined with endothelial protein C receptor (EPCR), regulates activated protein C, which inhibits factor V, factor VIII, and Plasminogen activation inhibitor (PAI-1). Beside its role of co-factor, TM has its own anticoagulant properties. ECs also express and release tissue plasminogen activator (t-PA), the main initiator of fibrinolysis.

The endothelium is the main regulator of the vasomotor tone *via* its capacity to produce and release vasoactive substances in response to several environment signals. Nitric oxide (NO) is the most important vasodilator agent and is constitutively produced by ECs. NO is generated by the endothelial nitric oxide synthase (eNOS) and derived from L-arginine. NO is constitutively released by ECs and eNOS is induced by chemical (ADP, bradykinin) or physical (shear stress) factors to adapt blood flow to various conditions. Then, NO applies its vasodilatory function, diffusing in vascular smooth cells to activate cyclic GMP production by guanylate cyclase. Moreover, besides its vasoactive action, NO possesses many other biological properties (e.g., anti-agregant, endothelial cell growth) contributing to vascular homeostasis.

## Endothelium In Sepsis

Because of their privileged contact with circulating blood, ECs are the first to interact with the microbial component and act as a “sentinel” for circulating micro-organisms. Moreover, the vascular endothelium is very sensitive to various stimulating factors that induce different phenotypes and initiate the immune response to infection.

ECs express Toll-like receptors (TLRs), surface receptors recognizing a pathogen antigen, such as Pathogen-Associated Molecular Patterns (PAMPs) and Damage-Associated Molecular Patterns (DAMPs). Some TLRs are ubiquitous whereas others depend on organs or are expressed under specific circumstances. Moreover, TLR expression and signalization can be modulated by inflammatory cytokines (8). ECs predominantly express TLR4, which is the main receptor for lipopolysaccharide (LPS), a Gram-negative bacteria molecule. However, TLR2 expression might also be triggered by inflammatory conditions. This recognition initiates intracellular signalization, resulting in expression of proinflammatory transcription factors such as Nuclear factor of the k-chain in B cells (NF-kB) (9).

### Endothelial Activation

During sepsis, activated ECs recruit immune cells (leukocytes) at the site of infection to eliminate the micro-organism and limit the spread of infection.

#### Proinflammatory Phenotype

NF-kB is a ubiquitous transcription factor involved in all major inflammatory reactions. It is implicated in cytokine production, molecule expression, cell survival, and differentiation (10). NF-kB activity can be induced in ECs by several stimuli, including inflammatory cytokines [Tumor necrosis factor (TNFα), Interleukin (IL-1)], and microbial components (LPS). Moreover, inhibiting NF-kB activity in mice stimulated by LPS led to decreased tissue neutrophilic infiltration and damage, suggesting its central role in sepsis (11).

During infection, ECs reprogram toward a proinflammatory and secretory phenotype. Numerous proteins produced by ECs are stocked in intracellular vesicles, the Weibel-Palade bodies. Under stimulation, ECs degranulate and release the content of these bodies (TF, P-selectin, vWF, angiopoietin-2) into the vasculature. ECs may then produce and release proinflammatory cytokines (IL-6) in the circulation, amplifying and spreading the inflammatory response in order to recruit immune cells at the infected site.

#### Pro-adhesive Phenotype

Activated endothelium and shedding of the glycocalyx in septic conditions lead to increased membrane expression of adhesion molecules that mediate leukocyte trafficking and recruitment to the area of infection. First, the selectins (E- and L-selectin) orchestrate the first phase of contact, rolling between circulating leukocytes and the endothelium. Then, prolonged and firm adhesion depends on immunoglobulin molecules, intercellular adhesion molecules (ICAM-1, ICAM-2), and the vascular adhesion molecule (VCAM). Finally, trans endothelial migration involves disruption of the tight junction and platelet endothelial cell adhesion molecule (PECAM). The final goal of this process is the diapedesis of leukocytes and extravasation into the tissues. The infiltration of immune cells in infected tissues is crucial to eliminate the pathogen. In experimental studies, LPS-exposed ECs expressed ICAM-1 and E-selectin mRNA, and blocking proinflammatory cytokines resulted in inhibition of mRNA transcription of adhesion molecules (12). Moreover, ECs release adhesion molecules in the circulation. E-selectin (13) and ICAM1 (14) plasma levels have been found to be increased in septic patients. The soluble circulating form of the adhesion molecules are increased in septic patients compared to patients admitted for a trauma (15), and the plasma levels of these adhesion molecules are closely related to sepsis severity (16).

#### Procoagulant Phenotype

In sepsis, ECs acquire procoagulant and antifibrinolytic properties which help to prevent dissemination of infection. Either expressed by activated immune cells or released from Webel-Palade bodies, TF level is increased in septic conditions. TF then initiates the coagulation cascade. In parallel, anticoagulant proteins [protein C, TFPI (17), TM] are downregulated by increased consumption and decreased production. Endothelial TM expression is decreased in sepsis and inactivated by leukocyte elastase release, limiting protein C activation. Skin biopsies of patients with purpura fulminans revealed a decreased expression of endothelial TM and ECPR in parallel with low blood levels of protein C, protein S and antithrombin (AT) (18). Moreover, decreased circulating activated protein C in neutropenic septic patients was associated with disease severity (19). In addition, the endothelium releases large amounts of PAI-1 when stimulated by IL-1 and TNF-α, resulting in an antifibrinolytic condition (20).

In addition, degradation of glycocalyx and EC apoptosis induce the release of large amounts of vWF. This permits the recruitment and aggregation of platelets at the infected site.

**Figure 1:**
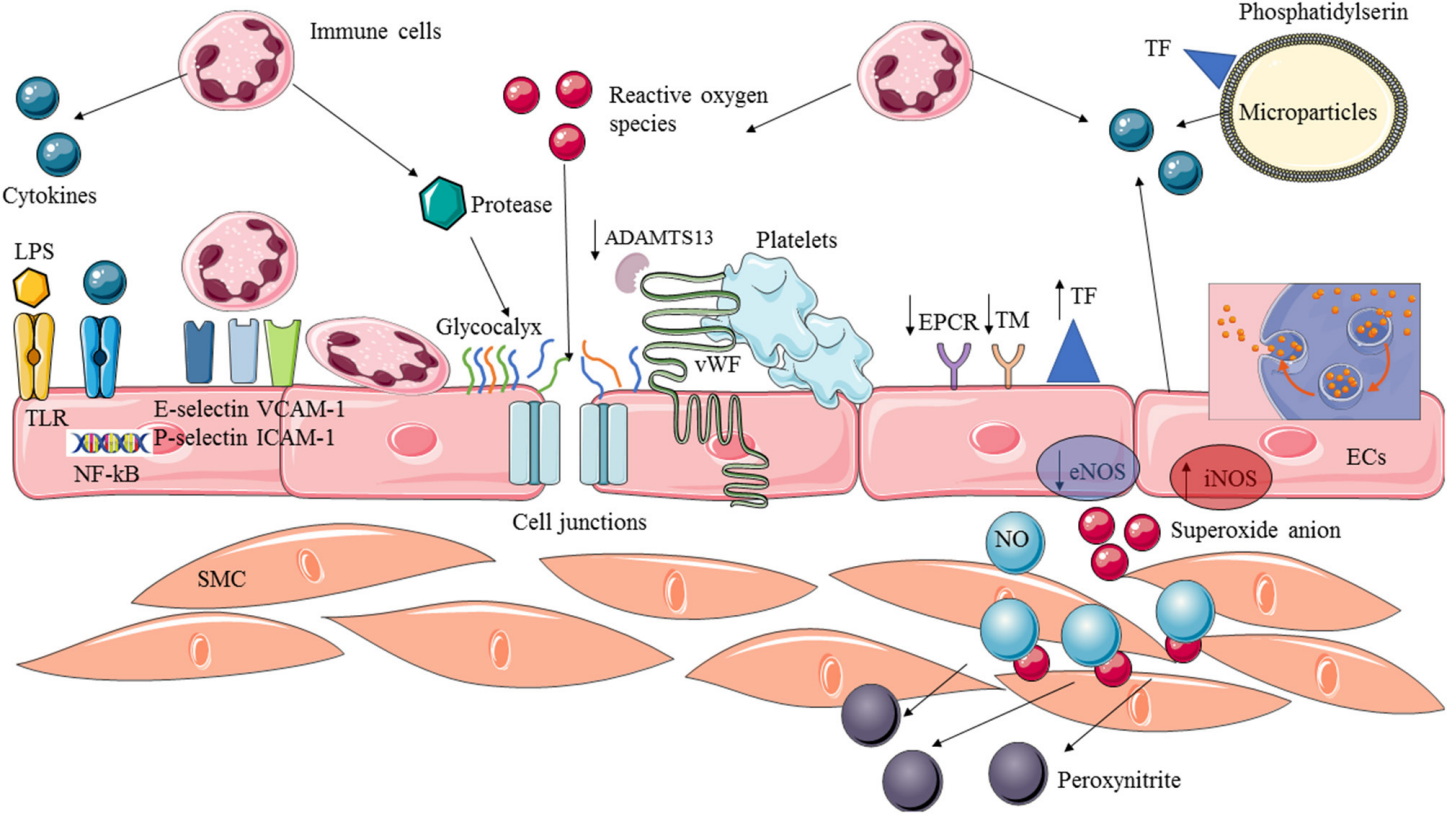
Endothelial activation and dysfunction in sepsis. Endothelial cells are provided with membrane receptors (ex:TLRs) and can be activated by various stimuli factors such as microbial components (LPS) or pro-inflammatory cytokines. The recognition of circulating stimuli leads to the activation of NF-kB transcription factor allowing expression of adhesion molecules and switch to a pro-inflammatory and pro-coagulant phenotype. Circulating leukocytes are recruited, adhere to the endothelium and extravasate into the tissue. Pro-coagulant protein are overexpressed whereas anticoagulant and profibrinolytic proteins are downregulated. Cytokines, ROS and protease released by activated immune cells can cause structural damage, leading to an increased permeability and exposition of endothelial-derived proteins. Vasoreactivity is impaired by (1) decreased production of NO by the eNOS, and (2) complexed available NO with superoxide anion to produce peroxynitrite. Moreover, ECs play a crucial role in spreading the inflammatory reaction which, in turn, produces cytokines and free radicals, and the release of microparticles. ADAMTS13, A disintegrin and metalloprotease with thrombospondin type I repeats-13; ECs, Endothelial cells; SMC, Smooth muscular cells; TF, Tissue factor; TM, Thrombomodulin; EPCR, Endothelial protein C receptor; vWF, Von Willebrand factor; LPS, Lipopolysaccharide; TLR, Toll like receptor; eNOS, Endothelial nitric oxide synthase; iNOS, Inducible nitric oxide synthase; NO, Nitric oxide; ICAM, Intercellular adhesion molecules; VCAM, Vascular cells adhesion molecules; NF-kB, Nuclear factor of k-chain of B.

Endothelium activation is therefore an appropriate and necessary response of the host to an acute infection. Thus, this microcirculatory response (leukocyte recruitment, coagulation) is adaptive and often successful in localizing and eliminating infectious insults (21). However, in extreme cases of overwhelming infection, these processes may contribute to overall morbidity, organ failure, and death. The challenge is in the balance between the adaptative immune response and endothelial activation to control the infectious process and excessive and maladaptive response. Moreover, this is a dynamic process and an adaptative response at one timepoint can become deleterious at another (Figure 1).

### Microvascular Endothelial Dysfunction

Endothelial dysfunction is defined by the loss of or exaggerated endothelial function.

The uncontrolled amplification of the host's proinflammatory response can lead to septic shock and the failure of distant, non-infected organs.

#### Structural Damage/Increased Permeability

In animal models of sepsis, ECs experienced morphological changes, including nuclear and cytoplasm lesion, rupture of cells, and even apoptosis. These kinds of damage have been confirmed in septic patients who demonstrate an increased number of circulating ECs and apoptotic bodies (22).

During sepsis, the damaged glycocalyx exposes a denuded endothelium. In an endotoxemic mouse model, the authors revealed pulmonary microvascular glycocalyx degradation related to TNF-alpha and heparinase. Interestingly, in this experimental study, inhibiting heparinase maintained glycocalyx integrity and reduced neutrophil adhesion and tissue damage (23). There are several consequences for the microcirculation following glycocalyx degradation, including decreased capillary density and increased permeability of macromolecules, although there are no macrohemodynamic changes (24).

In septic patients, glycocalyx impairment can be evidenced by the presence of glycocalyx shedding components in the circulation. The levels of circulating serum hyaluronan and syndecan are associated with the prognosis of septic patients (25). Moreover, new techniques can evaluate glycocalyx thickness in sublingual microcirculation. The decreased microvascular glycocalyx thickness in sepsis is correlated to microcirculation perfusion (26) and is an early predictor of mortality (27).

In addition, several pathways, like the Angiopoietin/Tie-2 (Ang/Tie2) axis, are altered during sepsis. Ang/Tie2 signaling is critical for maintaining vascular barrier integrity. During sepsis, the Ang/Tie2 system is disrupted, leading to increased microvascular permeability which contributes to organ failure and death (28). Moreover, the neutrophilic accumulation may enhance tissue and cell damage by generating inflammatory cytokines, reactive oxygen, and proteases (29).

All together, these structural damages imply disrupted barrier function and increased permeability, leading to interstitial leakage and edema. Moreover, the denuded endothelium and apoptotic ECs display a proinflammatory and procoagulant phenotype, enhancing the systemic response to infection. Furthermore, leukocyte adherence to the endothelium may participate in microvascular blood flow alterations (30).

#### Impaired Vasoreactivity

Microvascular endothelium is the chief regulator of vascular tone, mainly through the production of NO, a vasoactive soluble gas. During sepsis, NO production is dysregulated, with various changes depending on the course of the disease, leading to an impaired endothelium-dependent vasorelaxation and ultimately an alteration of microvascular blood flow.

First, there is a decreased production of NO by endothelial NO synthase, precipitating so-called “nitrosopenia”. The causes are multifactorial, with the downregulated expression of eNOS mRNA (31), a modified membrane receptor, and impaired signal transduction (32). Moreover, septic ECs are depleted of tetrahydrobiopterin, the limiting substrate for eNOS, uncoupling the enzyme and resulting in production of superoxide anion instead of NO.

The inducible NO synthase (iNOS) is also increased during sepsis (33). iNOS produces large amounts of NO, about 1,000-fold more than eNOS, and is responsible for intense and diffuse microvascular vasodilatation. This effect can cause an impaired response to norepinephrine. Moreover, the overproduced NO can complex with oxygen reactive species (superoxide anion) to generate peroxynitrite, a highly cytotoxic oxidant product. The rapid formation of peroxynitrite in an oxidative environment leads to reduced NO availability.

Overall, sepsis is characterized by a decrease of NO bioavailability and impaired vasoreactivity. Moreover, besides its effects on vascular tone, the lack of NO also results in dysregulated platelet adhesion and endothelium integrity. However, the pathophysiology of NO in sepsis is not fully understood as showed by the controversial therapeutic interventions targeting NO.

#### Oxidative Stress

Large amounts of reactive species are produced during sepsis, causing structural and cellular damage (41). There is an imbalance in the production of reactive species and a decrease in antioxidant agents. Firstly, oxidative species are produced in large amounts by inflammatory cells (42) and play a role in cell adhesion and inflammatory reaction (43). Besides being a target, ECs also produce and release reactive species (superoxide anion, hydrogen peroxide, radical hydroxyl) in sepsis through the NADPH pathway (44).

Inside cells, the accumulation of reactive species in the form of hydrogen peroxide and peroxynitrite causes protein and DNA damage, contributing to endothelial dysfunction (45). Moreover, besides complexing the NO to generate highly unstable peroxynitrite, oxidant agents induce uncoupling of eNOS (46), thus creating a vicious circle and amplifying oxidative stress (47). Overall, this results in decreased NO bioavailability. On the other hand, reactive oxygen and nitrogen species can enhance NF-kB activation and thus endothelial activation (48).

#### Microthrombi

Dysfunctional endothelium activates primary hemostasis and coagulation in a supra-physiologic way by decreased anticoagulant signaling. Generalized activation of coagulation during sepsis may enhance widespread microvascular injury (49, 50).

Autopsy studies of patients who died from *Streptococcus suis* infection found multiple microthrombi in the capillaries of the lung, kidney, and intestine (51). Several post-mortem studies have confirmed disseminated microthrombosis in the kidney, liver, brain, and gut microcirculation of septic patients (52).

Therefore, a new pathogenic entity in sepsis was defined as “endotheliopathy-associated vascular microthrombotic disease” involving microthrombosis mainly through unusually large vWF multimers (53). Indeed, sepsis may lead to acquired a disintegrin and metalloprotease with thrombospondin type I repeats-13 (ADAMTS13) deficiency since inflammatory mediators can inactivate ADAMTS13. ADAMTS13 is a metalloproteinase that cleaves the multimers of vWF in order to limit platelet activation in physiological conditions. Sepsis results in overexpressed vWF from ECs and a decreased availability of ADAMTS13. Then, large multimers of vWF recruit circulating platelets, thereby contributing to platelet-endothelium interactions (54, 55).

In parallel to leukocyte adherence to the endothelium (30), the presence of disseminated microthrombosis may participate in alterations to the microvascular blood flow (56). Platelet-to-endothelium adhesion is associated with stopped-flow capillaries and the inhibiting adhesion process may lead to an improved microvascular blood flow (57).

#### Microparticles

Microparticles (MPs) are small vesicles derived from activated or apoptotic cells which are released into the circulation with the detachment of cell membrane. MPs are embedded with various surface antigens. In physiological conditions, microparticles are essential to intercellular traffic and act as messengers. In response to an inflammatory environment, ECs release microparticles that can activate coagulation, amplifying the inflammatory and procoagulant response and dispersing it away from the initial site of infection. In sepsis, MPs express proinflammatory and procoagulant mediators. Endothelium-derived MPs expose phosphatidylserine at their surface, providing a privileged site to initiate coagulation cascade (58). Moreover, MPs express high levels of TF, which contribute to coagulation activation (59).

Delabranche and co-authors have suggested that circulating levels of endothelium-derived MPs in the plasma of septic patients may be a good predicting factor of outcome (60).

## Microcirculation In Sepsis

The microcirculation, composed of a series of <100 micron-diameter arterioles, venules and capillaries, is the terminus of the vascular tree (61). Arterioles mostly guarantee vascular tone because of their muscular surface, whereas capillaries provide oxygen delivery, nutriments, and solute exchange depending on the tissue need (61).

Sepsis is associated with profound changes in microcirculation (62).

### Microcirculatory Disorders

Microcirculatory alterations in sepsis mainly consist of a decreased capillary density and an increased heterogeneity of blood flow. As compared to hemorrhagic shock, endotoxemic shock leads to considerably greater impaired gut microvascular perfusion (63). During sepsis, microcirculation suffers from quantitative and qualitative alterations.

Firstly, sepsis is associated with a decrease in functional capillary density and a reduction in the proportion of perfused small vessels, whereas stopped and intermittently perfused capillaries are increased (64, 65). Altered capillary density has been observed in gut (64), brain (65), skin, and tongue (66) microcirculation in various septic animal models.

Sepsis-associated microcirculatory dysfunction is responsible for suboptimal capillary perfusion, resulting in impaired oxygen extraction by tissues, a finding which is not explained by impaired delivery of oxygen by systemic circulation (67). It has been shown that muscular microcirculation in a cecal ligation and puncture (CLP) rat model heterogeneously delivers oxygen to capillaries (68). Heterogeneous perfusion is defined by the presence of intermittently or non-perfused capillaries close to well perfused capillaries, and this process is dynamic over time. Minimal under physiological conditions, the heterogeneity of perfusion is highly increased in sepsis. Thus, heterogeneous perfusion exists between organs, with redistribution of blood flow to vital organs at the expense of other tissues, but it is also present within the same organ. Experimental studies evidenced microvascular shunting in sepsis, especially in the gut, creating local zones of hypoxia (69, 70). One of the main features of microvascular shunting can be attributed to the heterogeneity of perfusion (71).

The gut and splanchnic circulation represents an elective measurement site because of its early impairment in sepsis. Experimental studies provided evidence that the disturbance in oxygen extraction was related to the heterogeneity of microcirculatory perfusion. Heterogeneous perfusion is therefore considerably more deleterious than uniformly perfused capillaries regarding tissue oxygen extraction (72).

This heterogeneous perfusion also implies different expression of key proteins, enzymes, and molecules, which are increased during sepsis. For example, in endotoxemia rats, iNOS is differently expressed along the gut tractus, suggesting that the vasomotor tone within the gut is different (73). This observation has been confirmed in a sheep sepsis model in which animals were treated with iNOS inhibitors resulting in various microvascular blood flow responses in the gut, pancreas, and spleen (74).

Together, the decreased capillary density and heterogeneous alterations in microcirculation contribute to an increased diffusion distance for oxygen and an impaired extraction capacity, thus creating hypoxic zones. Heterogeneous expression of hypoxic genes within myocardial microcirculation has been demonstrated in a murine model of sepsis. Indeed, during entoxemia, Hypoxia inducible factor (HIF)-1a and Glucose transporter (GLUT)1 expression co-existed with ICAM-1 expression in malfunctioning capillaries, suggesting that microcirculatory alterations were associated with hypoxic zones (75).

## Association Between Microvascular Dysfunction And Outcome

Hemodynamic coherence is the hypothesis that normalization of systemic variables will be accompanied by improvement of microcirculatory perfusion in resuscitated patients (76). In experimental studies, microcirculatory disorders poorly correlate with systemic variables. Several authors have highlighted the loss of hemodynamic coherence in critically ill patients, especially in sepsis. The resulting consequences are: (1) conventional systemic parameters fail to reflect the state of microcirculation; (2) therapeutic strategies which aim to correct macrohemodynamic parameters are probably ineffective in improving microcirculation.

Knowing that microcirculatory disorders are central to the pathophysiology of sepsis, it is essential to assess their clinical relevance in the course of the disease and in patient outcome. Although macrohemodynamic parameters can be improved with conventional resuscitation interventions, the persistence of microcirculation abnormalities may explain unfavorable outcomes. Several experimental studies have found a correlation between survival and microcirculatory perfusion (77). Moreover, Zhang et al. provided strong evidence that micro- and macrocirculation are dissociated in endotoxemic shock, and that microcirculatory disorders precede the alterations in macrocirculation (78). Sakr and colleagues (3) demonstrated that, with equal baseline alterations, sublingual capillary density increased over the time course of septic shock in survivors, whereas this did not occur in non-survival patients. These microvascular alterations could predict ICU mortality contrary to global hemodynamic parameters. In a pilot observational study, the authors assessed sublingual microvascular perfusion by Sidestream Dark Field (SDF) in the early course of sepsis, with repeated measures during the early goal-directed therapy. They found that an improvement in microcirculatory perfusion during resuscitation was associated with less organ failure (79). Finally, De Backer et al. (80) confirmed the independence of micro- and macrovascular parameters and the strong association of sublingual microcirculatory parameters with outcome.

Although it is difficult to assess a causal relationship in the absence of randomized controlled trials, the development of more accurate techniques allows better identification of prognostic factors. Concordant data found several microcirculatory variables to be independent prognostic factors in sepsis and septic shock. Thus, the loss of hemodynamic coherence, and the association of microcirculatory disorders with outcome, support the extension of microcirculatory assessments and the use of microvascular variables to guide resuscitation.

## Microcirculatory Dysfunction Assessment

Microcirculatory dysfunction plays a crucial role in the pathophysiology of sepsis. Early recognition of critical illness severity, leading to an early appropriate resuscitation, is essential to improve outcome (1). For a long time, management of critically ill patients has focused on restoring systemic hemodynamic parameters. Indeed, patient monitoring has comprised assessment of macrohemodynamic parameters such as arterial pressure, and both urine and cardiac output. However, in the past few years, it has been shown that classic hemodynamic parameters fail to identify microcirculatory disorders. Beyond classic systemic monitoring, we need specific tools to assess microcirculation dysfunction (Table 1).

**Table 1:** Assessment of microcirculatory disorders in critically ill patients.

**Technique**	**Principle**	**Site of measure**	**Variable**	**Modifications in sepsis**	**References**
**Microvascular blood flow**					
Laser Doppler	Doppler effect Relative blood flow analysis	Skin, muscle, digestive mucosa	Blood flow (relative units)	Decreased blood flow Decreased reactivity	Eun et al. (81) Neviere et al. (82)
Pulsatility index	Doppler ultrasonography	Visceral organs (kidney, spleen, liver)	PI= (systolic blood flow velocity-minimum diastolic blood flow velocity)/mean blood flow velocity)	PI is increased in septic shock	Brunauer et al. (83)
**Videomicroscopy**					
Nailfold videomicroscopy	Transillumination Microscope with reflected light	Finger ungual microcirculation	Vascular density	*NA*	Freedlander et al. (84) Weinberg et al. (85)
OPS and SDF Cytocam incident dark field	Direct microvascular visualization Absorption of a selected wavelength by hemoglobin	Sublingual microcirculation	Capillary density, perfused capillary density, functional capillary density, microvascular flow index	Decreased capillary density Increased heterogeneity of perfusion	De Backer et al. (86) Dilken et al. (87) Aykut et al. (88)
**Tissue oxygenation**					
Near Infrared Spectrometry	Transmitted and reflected light at several wavelengths Different absorption properties of oxy- and deoxyhemoglobin	Muscle Thenar eminence	Tissue oxygen saturation: StO_2_ = (HbO_2_)/(HbO_2_+Hb)	Decreased StO_2_ Decreased reactivity	Mancini et al. (89) Creteur et al. (90)
PO_2_ electrodes	Transcutaneous electrodes that detect oxygen and carbon dioxide	Skin	Tissue PO_2_	*NA*	Vesterager (91)
**Peripheral tissue perfusion**					
Capillary refill time (CRT)	Return to baseline color of a soft tissue after applying a pressure for 15s	Fingertip or knee	Time (in seconds) to baseline return after releasing the pressure	Increased CRT	Ait-Oufella et al. (92) Lima et al. (93)
Mottling	Discoloration of the skin	Knee	Mottling extension	Increased mottling score	Ait-Oufella et al. (94) Ait-Oufella et al. (95)
Temperature gradient	Cutaneous probes to evaluate skin temperature	Skin, toe, finger	Central-to-skin temperature gradient Skin-to-room temperature gradient	High central-to-skin temperature gradient Reduced skin-to-room temperature gradient	Bourcier et al. (96)

*NA, not available; OPS, orthogonal polarization spectral imaging technique; O_2_, oxygen; PI, Pulsatility index; PO_2_, Partial oxygen pressure; SDF, sidestream dark field imaging technique; StO_2_, tissue oxygen saturation*.

### Microcirculatory Perfusion

Evidence for microcirculatory dysfunction was first found in experimental studies using intravital microscopy, allowing direct visualization of microcirculation with large microscopes on fixed tissues. Bedside assessment of microcirculation in critically ill patients is challenging, as intravital microscopy cannot be used in humans. The main concerns are the site of measurement, the timing (80), and the availability of validated tools. In the past decade, several techniques have been examined in the study of microcirculation.

#### Microvascular Blood Flow

The Laser Doppler flowmetry technique applies the Doppler effect, in which laser light shifts its frequency after reflecting from red blood cells. Laser Doppler provides a measure of relative microcirculatory blood flow defined by the average of the velocities in vessels in the area of interest. Thus, this technique does not give an absolute value of blood flow but a quantitative index of flux (81).

Laser Doppler flowmetry can be performed in skin, muscle, and mucosal microcirculation. Interestingly, gut mucosal perfusion is accessible to Laser Doppler using specific probes, and is of particular interest in critically ill patients to assess visceral organ perfusion (97).

This technique allows continuous microcirculatory blood flow recording and measurement of microvascular blood flow reactivity to various interventions (vascular occlusion test, pharmacologic products) (98). The method is advantageous in critically ill patients as it can be applied to the skin, an easily accessible site. It has been observed that basal blood flow is decreased in critically ill patients as compared to healthy volunteers. Moreover, vascular reactivity is impaired during sepsis (82).

The main limitation of this technique is the relatively large sample volume of analysis (0.5–1 mm^3^) in which arterioles, capillaries, and venules of diverse size and perfusion are analyzed within the same timeframe. In sepsis, where perfusion is particularly heterogeneous, even in the same area, this method may miss some perfusion abnormalities.

#### Pulsatility Index

The pulsatility index (PI) is used to estimate the organ vascular tone rather than to directly assess blood flow. The PI is assessed using a Doppler sonography technique which can explore visceral organs [kidney (99), spleen, liver (100)]. Using color Doppler, an artery of interest is identified and pulse wave Doppler is performed. The resulting blood flow signal is recorded, and PI is calculated as the ratio of systolic blood flow velocity-minimum diastolic blood flow velocity/mean blood flow velocity.

Brunauer and colleagues (83) reported alteration of the PI of the liver, spleen, intestine, and kidney in septic shock patients, which were correlated to peripheral perfusion alterations.

#### Tissue Oxygenation

Near infrared spectroscopy (NIRS) is a procedure which measures tissue oxygenation. It can reach vessels at 0 to 25 mm from the applied area. Limited to vessels of <1 mm diameter, NIRS focuses on microcirculation oxygenation (89).

In critically ill patients, the thenar eminence is the preferential site of measure, limiting confounding factors such as edema. Using transmitted and reflected light at different wavelengths, tissue oxygenation is estimated by the different absorption properties of oxy- and deoxyhemoglobin. Septic patients had a reduced tissular oxygen saturation (StO_2_) whose value correlated with organ failure and which was associated with poor outcome (101). This technique does not assess microvascular blood flow directly; however, it can be used to evaluate vascular reactivity. Septic patients evidenced reduced vascular reactivity, defined by a decreased slope in StO_2_ changes after occlusion challenge (90).

Microvascular partial oxygen pressure (PO_2_) electrodes can be used for direct tissue oxygenation assessment. This provides a reliable tissue PO_2_ in conditions of homogenous microcirculation (91). However, in the context of heterogeneous microcirculation, its accuracy decreases, limiting its use in critically ill patients.

#### Videomicroscopy

Nailfold videomicroscopy is historically the first procedure used in the clinical setting. It relies on the principle that organs can become translucent using reflecting light (84). The nailfold area provides an easily accessible, non-invasive site for direct visualization of the microcirculation under an ordinary microscope. Ungual microvascular blood flow undergoes severe impairment under various conditions (85). However, this technique has not been studied specifically in septic patients and is limited by its sensitivity to room temperature and local vasoconstriction.

Orthogonal polarization spectral imaging (OPS) and SDF are both techniques that rely on the principle of transillumination, which permits direct visualization of the microcirculation (86). The OPS light source emits polarized light which is reflected as: (1) (still) polarized light by the superficial layers, and (2) scattered depolarized light by the deeper layers of tissue which is absorbed by red blood cells. SDF uses green light and, like OPS, the superficial layer reflected light is not analyzed by the apparatus whereas reflected light from deep tissue reaches the center of the device. Red blood cells are seen as black bodies because of absorption of the light as a selected wavelength.

These two techniques allow direct visualization of the microcirculation with high contrast images provided by the reflected light from deeper layers. However, the visualization of microcirculation can be biased by the presence of red blood cells from the vessels.

Visualization has been mainly tested and validated in sublingual microcirculation because of the thin epithelial layer providing better images (87). The analyzed variables comprise vascular density, heterogeneity of perfusion, and capillary density (102).

In septic patients, De Backer et al. evidenced a decrease in the proportion of perfused small vessels (mainly capillaries) in addition to an increase in non or intermittently perfused capillaries (30).

These alterations are depicted even in the very early stages of sepsis. In addition, there are more severe sublingual microcirculatory alterations in non-survivor patients when compared to survivors (79).

One could question whether there is a correlation between sublingual microcirculation and vital organ perfusion. In pigs, sublingual and gut perfusion were similar during sepsis (103). Boerma and co-workers showed that sublingual microcirculation alterations are related to intestinal perfusion in septic patients (104). However, sublingual assessment presents some limitations (secretion, movement artifact), and cannot be used in patients with non-invasive ventilation.

These techniques use semi-quantitative analysis to perform microcirculatory evaluations and can only be used reliably by trained investigators (105). More recently, a third generation of handheld videomicroscope has been developed based on incident dark field imaging (IDF). Illuminating the vessel on all sides, IDF provides a three-dimensional effect and allows better optical resolution compared to SDF imaging (88). The last generation, Cytocam-IDF videomicroscope is a fully digitalized device computer-controlled, with a lower weight, high resolution sensor and a shortened pulse time. Therefore, IDF overcomes many of handheld microscopy previous limitations (106). Compared to SDF, Cytocam IDF provides superior image quality and better microcirculatory analysis (107) and in a preterm neonate's population, IDF imaging allowed visualization of 20 % more vessels than SDF (108).

The development of a bedside assessment of microcirculatory blood flow and oxygenation should provide a better and earlier recognition of microcirculatory dysfunction and guide resuscitation.

However, despite recent improvements, the application of these techniques is mainly confined to the field of research and rarely available in routine practice.

### Peripheral Tissue Perfusion Assessment

In critically ill patients, systemic hemodynamic parameters and biomarkers are not always an accurate reflection of microcirculatory disorders. The primary marker at the bedside is arterial lactate, which recognizes circulatory failure and guides resuscitation. However, hyperlactatemia is not specific to hypoperfusion and, in many cases, persistent hyperlactatemia is not related to circulatory dysfunction and can thus lead to over-resuscitation (92).

During septic shock, sympathetic activation redistributes blood flow toward the “noble organ” at the expense of less important tissue, such as the skin, which is deprived of autoregulation. Evaluation of perfusion of those tissues with non-invasive parameters can therefore provide a good estimation of microcirculatory disorders. Skin provides an easily accessible site at the bedside of critically ill patients.

#### Capillary Refill Time

The capillary refill time (CRT) is assessed by applying a pressure of 3 to 7 Newtons on the knee or on the fingertip for at least 2 s (93). There is agreement in the literature that “good” pressure is that needed to produce a “thin white distal crescent” under the physician's nail. After releasing the pressure, the time in seconds necessary to return the skin color to baseline defines CRT. It provides an easy-to-use clinical tool to assess skin perfusion and microcirculatory dysfunction. In trained physicians using standardized pressure and chronometer time recording, CRT has a good reproducibility (109).

This quantitative tool can reliably provide information on critical illness severity. Lima et al. demonstrated that a prolonged fingertip CRT longer than 4.5 s is associated with high arterial lactate level and organ failure in critically ill patients (110). In septic patients, Brunauer and colleagues showed that CRT is related to the PI (83). Moreover, the persistence of prolonged CRT (>2.4 s at the fingertip and >4.9 s at the knee) after resuscitation of septic shock patients is a reliable predictive factor of 14-day mortality (109).

Recently, it has been suggested that CRT could guide resuscitation therapeutics. The ANDROMEDA-SHOCK study (111) compared a fluid resuscitation initiation and cessation guided by arterial lactate or by CRT. The authors found no significant difference in primary outcome between the groups. However, this study suggests that a resuscitation strategy based on CRT can decrease fluid and vasopressor administration. Moreover, the latest guidelines from the Surviving Sepsis Campaign recommends taking into account CRT measurement in the management of septic shock patients (96).

#### Skin Temperature

The aspect of the skin in circulatory failure circumstances has historically been described as “pale and cold” (94). Using probes, skin temperature is easily accessible in critically ill patients. Septic shock patients experience increased central-to-skin and decreased skin-to-room temperature gradients. This provides quantitative information which is related to ICU mortality (95).

#### Mottling

Mottling is described as a purple discoloration of the skin, primarily localized in the knee area. Although the pathophysiology of mottling is not completely understood, it is related to alterations of skin perfusion (112). Ait-Oufella et al. provided a semi-quantitative evaluation score of mottling which depended on skin extension of mottling around the knee area. This score can reliably predict sepsis severity and mortality (113).

Mottling provides an excellent risk stratification tool. This is a non-invasive and easily used bedside tool, with excellent reproducibility even in non-trained clinicians. In septic shock patients, the mottling score is correlated to organ failure and is a strong predictor of 14-day mortality (114). De Moura et al. confirmed the excellent positive predictive value of a mottling score of 4 or more to predict 28-day mortality (115).

A multimodal approach taking into account these markers should be recommended for personalized management of septic patients. Lavelligrand et al. showed in a retrospective observational study that daily clinical evaluation, including the mottling score, oliguria, lactatemia, and neurological exam, may allow physicians to tolerate a mild arterial hypotension in septic patients (116).

Peripheral perfusion assessment suffers from several limitations. First, CRT and mottling are difficult to assess in patients with dark skin. Second, due to the high heterogeneity of microcirculation in organs during sepsis, these markers may not accurately reflect visceral organ perfusion.

## Therapeutic Axes

Sepsis and septic shock represent one of the leading causes of critically ill mortality despite improvement in management (early antibiotic therapy, fluid resuscitation, vasopressors). Alterations in microcirculatory perfusion play a key role in the pathophysiology of sepsis and are associated with organ failure. Resuscitating the microcirculation should be considered a major therapeutic goal in septic patients (117). Considering the loss of hemodynamic coherence, *one* of the foremost questions is whether the most common therapeutics used to resuscitate macrohemodynamic parameters impact the microcirculation.

### Fluid Resuscitation

Fluid resuscitation is the administration of fluids (crystalloids, colloids) in hypotensive patients to restore organ perfusion. However, there is some debate about the microcirculatory consequences. In an experimental study, fluid resuscitation worsened shock severity and impaired the microcirculation (118). Conversely, Santos et al. found an improvement of capillary density and blood flow following fluid resuscitation in a rodent model (77). In humans, Ospina-Tascon and team (119) found that fluid administration might improve sublingual microvascular perfusion for patients in the early sepsis phase. However, this effect was not confirmed in later phases of sepsis. Interestingly, the authors demonstrated a dissociation between micro- and macrohemodynamic response to fluid resuscitation in some patients with no microvascular response despite improvement of cardiac output. Moreover, the type of fluid used, whether colloids or crystalloids, had no impact on sublingual microcirculation in this study (120). Recently, in a pilot study of 35 septic shock patients, Hariri et al. (121) found an improvement in skin microvascular endothelial function in patients receiving albumin compared to crystalloids.

### Inotropes/Vasopressors

Vasopressive and inotropic agents are used in sepsis to maintain organ perfusion pressure. However, their effects on microcirculation may vary among patients and between different organs (122). Experimental studies have suggested that dobutamine might improve hepatic (123) and mesenteric (124) but not renal perfusion (125). Using the OPS imaging technique, De Backer et al. additionally showed that administration of dobutamine partially recruited the microcirculation independently of systemic hemodynamic parameters in the early phase of sepsis. However, this effect was not maintained in the later phases of the disease (126). In a randomized controlled trial, dobutamine failed to improve sublingual, peripheral, and splanchnic perfusion in septic shock patients, when compared to placebo (127). Conversely, Morelli and team (128) found that, due to its vasodilatory effects, levosimendan significantly improved microcirculatory blood flow and increased perfused capillary density in septic shock patients, as compared to dobutamine.

Similarly, data focusing on the microvascular effects of vasopressor agents are controversial. While norepinephrine improved both hepatic (129) and renal blood flow (130) in endotoxemic shock, this observation was not confirmed in another study focusing on the liver (131) and gut (132) microcirculations. In septic shock patients, norepinephrine failed to improve sublingual microvascular blood flow despite restoring macrohemodynamic parameters (mean arterial pressure) (133). Indeed, increasing blood pressure in septic patients did not affect various microcirculatory and perfusion parameters (134). Due to the complex pathophysiology of sepsis, which implies an imbalance between vasodilatation and vasoconstriction, it is reasonable to expect that the microvascular response might be highly unpredictable.

Microhemodynamic parameter assessment is required to evaluate the effects of the main drugs used during sepsis, as well as to select the patients for randomized trials in a tailored strategy, taking into account both macro- and microhemodynamic parameters.

Many strategies targeting endothelial and microvascular dysfunction have been proposed. Therapeutic drugs focus on endothelial vasoreactivity, inflammation, oxidative stress, and coagulation/anticoagulation balance. Table 2 (34–40) presents the major recent trials focusing on microcirculation during sepsis. Overall, while some studies showed an improvement in microcirculatory parameters, very few interventions succeeded in reducing mortality in critically ill septic patients. As endothelial activation is part of the host's required response during sepsis, its inhibition may be deleterious. Conversely, targeting one pathway may not be sufficient to improve outcome, and combination therapy should be discussed for future trials.

**Table 2:** Human randomized controlled trials targeting endothelium and microcirculation in sepsis and septic shock.

**Tested intervention**	**Study**	**Study design**	**Microcirculatory assessment**	**Primary outcome**	**Results/status**
**Oxidative stress**					
**Vitamin C**	The Vitamin C, Hydrocortisone and Thiamine in Patients With Septic Shock Trial (VITAMINS) (34)	*216 patients* High dose Vitamin C (6 g/d), Thiamine (400 mg/d) and Hydrocortisone (200 mg/d) vs. Hydrocortisone alone	NA	Vasopressor-free days	Not significant
	Hydrocortisone, vitamin C, and thiamine for the treatment of sepsis and septic shock (HYVCTTSSS) [NCT03258684]	*80 patients* Vitamin C 1.5 g/6 h, hydrocortisone 50 mg/6 h and thiamine 200 mg/12 h vs. saline	NA	Hospital mortality	Completed Awaiting results
	Metabolic Resuscitation Using Ascorbic Acid, Thiamine, and Glucocorticoids in Sepsis (ORANGES) [NCT03422159]	*140 patients* Vitamin C (4 g/d), Hydrocortisone (200 mg/d), thiamine (400 mg/d) for 4 days vs. placebo	NA	– Time to vasopressor independence – Change in SOFA score at day 4	Completed Awaiting results
	Vitamin C and Thiamine in Sepsis [NCT03592277]	*120 patients* Vitamin C (4 g/d) and Thiamine (400 mg/d) vs. placebo	NA	60-day mortality	Recruiting
	Ascorbic acid, Corticosteroids, and thiamine in sepsis (ACTS) trial [NCT03389555]	*205 patients* Vitamin C 1.5 g/6h, Thiamine 100 mg/6 h and Hydrocortisone 50 mg/6 h for 4 days vs. placebo	NA	SOFA score at 72 h	Completed, not published
	Clinical trial of antioxidant therapy in patients with septic shock [NCT03557229]	*131 patients* Vitamin C 4g/d, vitamin E 1,200 UNT/d, N-acetyl cysteine 2,400 mg/d and melatonin 50 mg/d for 5 days vs. placebo	Oxidative stress and inflammatory biomarker	SOFA score at day 7	Not yet recruiting
	Evaluation of Hydrocortisone, Vitamin C and Thiamine for the Treatment of Septic Shock (HYVITS) [NCT03380507]	*106 patients* Vitamin C 6 g/d, hydrocortisone 200 mg/d and thiamine 400mg/d vs. placebo	NA	60-day mortality	Terminated due to futility
	Ascorbic acid and thiamine effect in septic shock (ATESS) [NCT03756220]	*116 patients* Vitamin C 100 mg/kg/d, Thiamine 400 mg/d vs. placebo	NA	Change in SOFA score at 72 h	Completed Awaiting results
**Para-tyrosine**	Efficacy of Para-Tyrosine Supplementation on the Survival and Clinical Outcome in Patients With Sepsis [NCT03278730]	*296 patients* Para-tyrosine 2 g x 3/d oral or enteral for 7 days vs. placebo	NA	30-day mortality	Not yet recruiting
**Melatonin**	Efficacy of Melatonin in Patients With Severe Sepsis or Septic Shock [NCT01858909]	*110 patients* 30 mg/12 h 28 days vs. placebo	Oxidative stress and inflammatory biomarkers	28-day mortality and organ dysfunction	Unknown status
	Effects of Melatonin as a Novel Antioxidant and Free Radicals Scavenger in Neonatal Sepsis [NCT03295162]	*55 patients* Melatonin 10 mg x 2/days vs. placebo	NA	Free radicals scavenge	Awaiting results
**Inflammation**					
**Evolocumab (anti-PSCK9)**	Evolocumab for PCSK9 Lowering in Early Acute Sepsis (The PLEASe Study) [NCT03869073]	*36 patients* Low dose (420 mg) vs. high dose (840 mg) vs. placebo in 3 SC injections	NA	Decrease bacteria LPS levels	Recruiting
**Atorvastatin**	Study the Impact of Statins in Septic Shock [NCT02681653]	*80 patients* Atorvastatin 40 mg/d or placebo for 7 days	Cytokines	28-day mortality	Awaiting results
**Nitric oxide/Vasoreactivity**					
**NOS inhibitor**	Nitric oxide synthase inhibitor 54C88 in patients with septic shock (35)	*797 patients* NOS inhibitor vs. placebo for 7 or 14 days	NA	Mortality	Increased mortality in intervention group
**NO**	Randomized controlled trial of inhaled nitric oxide for the treatment of microcirculatory dysfunction in patients with sepsis (36)	*49 patients* Inhaled NO 40 parts per million for 6 h vs. placebo	Sublingual microcirculation using sidestream dark field videomicroscopy	– Change in SOFA score – Change in sublingual microcirculation flow index	Not significant
**Nitroglycerin**	Effects of nitroglycerin on sublingual microcirculatory blood flow in patients with severe sepsis/septic shock after a strict resuscitation protocol: a double-blind randomized placebo controlled trial (37)	*70 patients* Nitroglycerin at 4 mg/h for 30 min then 2 mg/h for 24 h or placebo	Sublingual microcirculatory blood flow using SDF imaging	Sublingual microcirculatory flow index	No significant differences
**Methylene blue**	Effect of methylene blue on hemodynamic and metabolic response in septic shock patients (38)	*64 patients* Methylene blue vs. placebo	NA	Septic shock resolution	Unknown status
	Methylene Blue in Early Septic Shock (SHOCKEM-Blue) [NCT04446871]	*91 patients* I.V. infusion of 100mg of methylene blue for 6 hours x3 doses a day vs. placebo	NA	Vasopressor requirement	Awaiting results
	Methylene Blue and Microcirculation in Septic Shock [NCT04295993]	*32 patients* Methylene blue 2 mg/kg over 10 min vs. standard of care	Sublingual microcirculation	Microvascular flow index at 6 hours	Not yet recruiting
**Ilomedin**	Ilomedin in Septic Shock With Persistent Microperfusion Defects (I-MICRO) [NCT03788837]	*235 patients* I.V. ilomedin at 0.5 ng/kg/min with increments of 0.5 ng/kg/min every 30 min up to a maximum of 1.5 ng/kg/min for 48 h vs. placebo	Mottling, cutaneous laser Doppler, NIRS, videomicroscopy, tissular PCO_2_, perfusion index	Change in SOFA score at day 7	Recruiting
	Co-administration of Iloprost and Eptifibatide in Septic Shock Patients (CO-ILEPSS) [NCT02204852]	*18 patients* Iloprost 1 ng/kg/min + Eptifibatide 0.5 ug/kg/min for 48 h continuously vs. placebo	Biomarkers of inflammation, coagulation, adhesion molecules	Biomarkers of endothelial activation and dysfunction	Awaiting results
	Infusion of Prostacyclin (Iloprost) vs. Placebo for 72-hours in Patients With Septic Shock Suffering From Organ Failure (COMBAT-SHINE) [NCT04123444]	*380 patients* Continuous infusion of 1 ng/kg/min for 72 h vs. placebo	NA	Change in SOFA score	Recruiting
**Citrulline**	Citrulline in Severe Sepsis [NCT01474863]	*72 patients* Low dose vs. high dose vs. placebo	NA	Vasopressor dependency index	Not published
	Effect of Citrulline on the Clinical and Biochemical Evolution of Patients With Sepsis. (CITRUSEP) [NCT02370030]	*176 patients* Citrulline malate 10g/day for 7 days vs. placebo	NA	Multiple organ failure	Unknown status
***Coagulation/hemostasis***					
**Thrombomodulin**	Effect of a recombinant human soluble thrombomodulin on mortality in patients with sepsis-associated coagulopathy (SCARLET study)	*800 patients* I.V. thrombomodulin at 0.06 mg/kg/d vs. placebo for 6 days	Biomarkers	28-day mortality	Not significant
**Protein C**	Drotrecogin Alfa (Activated) in Adults with Septic Shock (39)	*1,697 patients* Drotrecogin alfa or placebo for 96 hours	NA	28-day mortality	Not significant
	Human protein C concentrates in patients with sepsis and septic shock [NCT01411670]	*60 patients* with protein C activity <60% Human Protein C concentrate of activated protein C vs. placebo	Sublingual microcirculatory blood flow	Sublingual microcirculatory blood flow assessed by SDF	Awaiting results
	Modulation of vasoreactivity in septic shock: impact of recombinant protein C [NCT02885168]	*30 patients* Recombinant activated protein C during 96 h vs. placebo	Near-infrared spectroscopy with reactive hyperemia	Vascular reactivity	Awaiting results
**Recombinant antithrombin**	Recombinant human Antithrombin (ATryn) in the treatment of patients with DIC associated with severe sepsis [NCT00506519]	*25 patients* High dose or low dose of I.V. antithrombin vs. placebo for 5 days	Inflammatory markers	Improvement in the DIC score by 2 points at day 28	Awaiting results
**Antithrombin** **+** **recombinant human thrombomodulin**	The efficacy and safety of antithrombin and recombinant human thrombomodulin combination therapy in patients with severe sepsis and disseminated intravascular coagulation (40)	*129 patients* Antithrombin + thrombomodulin vs. antithrombin alone	NA	Platelet count and D-dimer levels at day 7	Intervention group had significant improvement of platelet count and D-dimer levels at day 7
**Heparin**	Efficacy and Safety of Unfractionated Heparin on Severe sepsis With Suspected Disseminated Intravascular Coagulation [NCT02654561]	*700 patients* Heparin 12,500 units/d for 7 days	NA	ICU mortality	Recruiting
	Heparin Anticoagulation to Improve Outcomes in Septic shock: The HALO Pilot [NCT01648036]	*76 patients* Unfractionated heparin 18 UI/kg/h continuous I.V. for 7 days vs. Dalteparin 5000IU subcutaneous daily	Biomarkers	Unknown	Not published
**Annexin 5**	SY-005(Recombinant Human Annexin A5)in Patients With Sepsis [NCT04898322]	*96 patients* Annexin 5 vs. placebo for 5 days	Biomarkers	Safety and tolerability	Not yet recruiting
**Aspirin**	ASpirin for Patients With SEPsis and SeptIc Shock (ASP-SEPSIS) [NCT01784159]	*240 patients* 200 mg/day of placebo for 7 days	NA	Change in SOFA score at day 7	Recruiting
***Heart rate control***					
**Landiolol**	LANdiolol MIcrocirculatory Effects During Septic shOck (MILANOS) [NCT04931225]	Landiolol (Rapibloc) perfusion will be started at T0 at 0.5 mcg/kg/min and increased by 0.5 mcg/kg/min every 30 min in order to achieve a 15% (T1) decrease in heart rate	Laser Doppler coupled with iontophoresis of acetylcholine	Microcirculatory reactivity	Not yet recruiting
**Ivabradine**	Ivabradine for Heart Rate Control In Septic Shock (IRISS) [NCT04031573]	*429 patients* Ivabradine 2.5 to 7.5 mg/12 h *via* enteral administration, to achieve a heart rate of 80–94 bpm vs. placebo	NA	– Succeed in heart rate control – 28-day mortality	Recruiting

*d, day; DIC, Disseminated intravascular coagulation; h, hours; ICU, Intensive care unit; I.V., intravenous; LPS, Lipopolysaccharide; NIRS, Near Infrared Spectrometry; NA, not available;NO, Nitric Oxide; NOS, Nitric oxide synthase; PCO2, partial pressure of carbon dioxide; SDF, Sidestream Dark Field; SOFA, Sequential Organ Failure Assessment; SC, subcutaneous*.

Given the encouraging results in recent studies and incoming trials, new therapeutic targets deserve attention.

In the past few years, many authors have evidenced a decrease of antioxidant defense in septic patients (135). Vitamin C, or ascorbic acid, is a well-known antioxidant molecule, easily accessible and safe to use at the bedside (136). Several trials have studied the combination of vitamin C, thiamine, and hydrocortisone in septic shock patients. A meta-analysis of nine randomized controlled trials confirmed that this combination therapy could improve Sequential Organ Failure Assessment (SOFA) score and vasopressor-free days. However its benefit for survival is still under debate (137). Recently, Lavillegrand et al. found that supplementation of vitamin C in septic shock patients with persistent peripheral tissue impairment improved skin microvascular reactivity and peripheral perfusion in patients with or without vitamin C deficiency (138).

Other studies have focused on the adrenergic system. Indeed, the deleterious effects of sympathetic overstimulation in septic shock led several authors to study beta blockers. Morelli and co-workers (139) studied esmolol in order to reduce heart rate in septic shock patients. The authors found that the use of esmolol was safe, and improved outcomes of septic shock patients, and that it also improved microvascular blood flow (140). These results led to other trials that aimed to reduce heart rate and adrenergic stress in septic patients (Table 2).

## Conclusion

In the past two decades, the endothelium has been the focus of particular interest, especially in sepsis. As the major regulator of vascular homeostasis, the endothelium is one of the leading actors in response to aggression. In sepsis, the exaggerated and systemic endothelial activation leads to microcirculatory alterations which thus participate in organ failure and death. Recent advances in the assessment of the microcirculation promote a better understanding of microcirculatory impairments in sepsis. However, their use in clinical practice is limited by their availability and difficulty of use in critically ill patients with multiple confusing factors. New technical devices and clinical tools can be useful at the bedside to recognize microcirculatory impairments.

Despite improvements in patient care, sepsis and septic shock lead to high morbidity and mortality in critical care. Testing the prognostic value of microcirculatory disorders in sepsis, several trials have studied new molecules targeting endothelial functions and dysfunctions. However, despite some promising leads, the foremost studies have shown unfavorable results for outcomes of mortality or organ failure. We believe that microcirculatory resuscitation should be one of the goals in the management of septic patients. It is therefore crucial to identify microvascular endothelial dysfunction more effectively to better select patients for future trials.

## Author Contributions

LR and LZ both performed the review of the literature and wrote the study. All authors contributed to the article and approved the submitted version.

## Conflict of Interest

LZ has received a research grant from Jazz Pharmaceuticals, not related to this study. The remaining author declares that the research was conducted in the absence of any commercial or financial relationships that could be construed as a potential conflict of interest.

## Publisher's Note

All claims expressed in this article are solely those of the authors and do not necessarily represent those of their affiliated organizations, or those of the publisher, the editors and the reviewers. Any product that may be evaluated in this article, or claim that may be made by its manufacturer, is not guaranteed or endorsed by the publisher.

## Abbreviations

Ang/Tie2: Angiopoietin-1/Tie2 receptor
APC: Antigen-presenting cell
AT: Antithrombin
CLP: Cecal ligation and puncture
CRT: Capillary refill time
DAMPs: Damage-Associated Molecular Patterns
ECs: Endothelial cells
EPCR: Endothelial protein C receptor
GLUT 1: Glucose transporter
HIF: Hypoxia inducible factor
ICU: Intensive Care Unit
IDF: Incident dark field imaging
IL-1: Interleukin
iNOS: Inducible NO synthase
LPS: Lipopolysaccharide
MPs: Microparticles
NF-kB: Nuclear factor of the k-chain in B-cells
NIRS: Near infrared spectroscopy
NO: Nitric oxide
eNOS: Endothelial nitric oxide synthase
OPS: Orthogonal polarization spectral imaging
O2: Oxygen
PAI-1: Plasminogen activation inhibitor 1
PAMPs: Pathogen-Associated Molecular Patterns
PECAM: Platelet endothelial cell adhesion molecule
PI: Pulsatility index
PO2: Partial oxygen pressure
SDF: Sidestream dark field
SOFA: Sequential Organ Failure Assessment
StO2: Tissular oxygen saturation
t-PA: Tissue plasminogen activator
TF: Tissue factor
TFPI: Tissue factor pathway inhibitor
TLR: Toll-like receptor
TM: Thrombomodulin
TNF-α: Tumor necrosis factor
VCAM: Vascular adhesion molecule
vWF: Von Willebrand factor.
